# Automatic pavement texture recognition using lightweight few-shot learning

**DOI:** 10.1098/rsta.2022.0166

**Published:** 2023-09-04

**Authors:** Shuo Pan, Hai Yan, Zhuo Liu, Ning Chen, Yinghao Miao, Yue Hou

**Affiliations:** ^1^ Beijing Key Laboratory of Traffic Engineering, Beijing University of Technology, Beijing 100124, People's Republic of China; ^2^ National Center for Materials Service Safety, University of Science and Technology Beijing, Beijing 100083, People's Republic of China; ^3^ Department of Civil Engineering, Faculty of Science and Engineering, Swansea University, Swansea SA1 8EN, UK

**Keywords:** pavement detection, deep learning, convolutional neural network, Siamese network, one-dimensional convolution

## Abstract

Texture is a crucial characteristic of roads, closely related to their performance. The recognition of pavement texture is of great significance for road maintenance professionals to detect potential safety hazards and carry out necessary countermeasures. Although deep learning models have been applied for recognition, the scarcity of data has always been a limitation. To address this issue, this paper proposes a few-shot learning model based on the Siamese network for pavement texture recognition with a limited dataset. The model achieved 89.8% accuracy in a four-way five-shot task classifying the pavement textures of dense asphalt concrete, micro surface, open-graded friction course and stone matrix asphalt. To align with engineering practice, global average pooling (GAP) and one-dimensional convolution are implemented, creating lightweight models that save storage and training time. Comparative experiments show that the lightweight model with GAP implemented on dense layers and one-dimensional convolution on convolutional layers reduced storage volume by 94% and training time by 99%, despite a 2.9% decrease in classification accuracy. Moreover, the model with only GAP implemented on dense layers achieved the highest accuracy at 93.5%, while reducing storage volume and training time by 83% and 6%, respectively.

This article is part of the theme issue 'Artificial intelligence in failure analysis of transportation infrastructure and materials'.

## 1. Introduction

Pavement texture is a vital road characteristic with notable effects on skid resistance [1,2], tire-pavement noise [3,4] and road maintenance [5,6]. Changes in pavement texture during road use may lead to potential traffic safety risks and affect the quality of road service. Thus, accurate recognition of pavement texture is an essential prerequisite for performing road maintenance. However, the complexity of factors affecting the appearance of pavement texture may pose challenges to identifying pavement texture types promptly and accurately in many situations. As a result, large-scale pavement texture recognition tasks typically require substantial human and financial resources.

Deep learning has gained remarkable attention due to its ability to extract target features and learn iteratively using convolutional neural networks (CNNs) in various fields, including autonomous driving [7], materials [8], medicine [9] and road detection [10]. Specifically, for pavement texture recognition, deep learning has shown excellent performance. Chen *et al*. [11] applied the Dense Convolutional Network to classify pavement texture by generating adversarial networks to expand pavement texture, demonstrating the superiority of deep learning over manual classification and traditional machine learning methods. Liu *et al*. [12] proposed a precise and stable framework for pavement texture measurement and reconstruction, using a depth CNN-based encoder to extract features from pavement images and converting them into models using feature mapping units and decoders. Dong *et al*. [13] proposed an effective encoder–decoder CNN architecture based on the residual network for the reconstruction of pavement texture, which proved to be effective for three-dimensional feature analysis. Yoo *et al*. [14] introduced a novel method of pavement classification based on deep learning by using the tire-pavement interaction noise, which reflects the surface contour and texture properties of the road, as the data source. Chhay *et al*. [15] proposed a faster region-based CNN based on deep learning to count the exposed aggregate number representing texture wavelengths on digital images of exposed aggregate concrete pavement.

Although deep learning methods have demonstrated impressive classification accuracy in various tasks, the requirement for substantial amounts of training data often poses a challenge in real-world engineering applications. Few-shot learning has emerged as a promising alternative to overcome this obstacle, as it enables effective classification tasks with limited data availability. Several network architectures, such as Siamese networks [16,17], matching networks [18], prototype networks [19] and relation networks [20], have been designed and implemented specifically for few-shot learning applications. Numerous studies have demonstrated satisfactory results in classification tasks using few-shot learning techniques, even with fewer than 10 samples available for training each classification category. This highlights the potential of few-shot learning as a viable solution for tackling data scarcity issues in various domains. For example, Wang *et al*. [21] proposed a multi-attention mutual information distributed framework that combines distributed learning with few-shot learning to reduce time consumption and achieve desired results in the evaluation. Hu *et al*. [22] integrated unsupervised descriptor selection into few-shot learning, which employs a descriptor selection module to select and localize semantically meaningful regions in feature maps without supervision, thereby improving the efficiency of task adaptation and classification performance. Singh *et al*. [23] introduced dual class information, base class label and self-supervised class label for the base class training images to help the model learn generic features and improve the novel class performance in few-shot learning. Zeng *et al*. [24] developed a self-attention and mutual-attention module to learn feature correlations and reduce background interference in few-shot learning classification of remote-sensing images, which led to higher accuracy. Dong *et al*. [25] used the back-end network to extract multilevel feature information from the base class and improved the loss function to maximize the distance between different categories and minimize the distance between the same categories in few-shot learning. Guo *et al*. [26] introduced the pairwise similarity module into few-shot learning and generated the calibrated class centres suitable for the query sample by extracting the semantic correlations between the support and query sample and enhancing the discriminant regions. It is clear that few-shot learning has great potential to address pavement texture recognition issues.

Road maintenance professionals tend to use mobile devices in the field due to their portability, which limits the storage capacity of embedded models. Additionally, rapid feedback of classification results is often needed in engineering practice. Therefore, it is important to develop lightweight few-shot learning models applicable to practical engineering scenarios. Reducing the time and computational cost of deep learning models can bridge the gap between theoretical models and practical applications in many fields, and a number of lightweight methods have been proposed to this end. Sun *et al*. [27] proposed a lightweight network architecture for image super-resolution, using depthwise separable convolution to enhance the efficiency of the convolution operation and stacked weighted multi-scale residual blocks to improve the representation capability. Jiang *et al*. [28] developed a method for network compression in super-resolution, which employed weight pruning and a multi-slicing network of information to extract and integrate multi-scale features. Grassucci *et al*. [29] introduced a family of parameterized hypercomplex neural networks that directly captured convolution rules and filter organization from data, making them flexible in any user-defined or tuned domain. Cheng *et al*. [30] proposed a lightweight unified fusion network for multi-focus image fusion, which used guided filtering to separate the source image into basic and detailed layers and applied a gradient perception strategy to handle the fusion problem. Liu *et al*. [31] developed a lightweight generative adversarial network that used squeeze and expand, multi-scale convolution, and depthwise separable convolution to reduce network parameters and training time. Gong *et al*. [32] introduced a global contextually guided lightweight network that employed secondary cross-modal integration to remove redundant information and added a hybrid feature-cascaded aggregation module to emphasize the global context, along with calibration. However, despite the potential of few-shot learning for pavement texture classification, the development of lightweight few-shot learning models specifically for this application has not been extensively investigated, presenting an important research gap.

This study proposes a pavement texture classification model based on few-shot learning, aiming to improve its suitability for engineering practice by making it lightweight. The dataset used was constructed with three-dimensional laser scanning pavement texture data. The Siamese network is chosen as the basic framework to construct the original few-shot learning model, considering the specific characteristics of the pavement texture data. To reduce the parameter quantity and multiply-accumulate operations, lightweight methods such as global average pooling (GAP) and one-dimensional convolution are employed in different parts of the few-shot learning model. Comparative experiments were conducted to evaluate the performances of the lightweight models and the original few-shot learning model. The applicability of the proposed models in actual engineering tasks was then discussed based on the results. This study contributes twofold: (i) verifying the feasibility of few-shot learning on four-way five-shot pavement texture classification and (ii) proposing specific lightweight pavement texture classification models, balancing accuracy with storage volume and training time. By focusing on the practical requirements of pavement texture recognition in the field of road maintenance and addressing the limitations of current deep learning models, this study aims to provide valuable insights for practitioners and researchers alike. The proposed lightweight few-shot learning models have the potential to significantly impact the efficiency and effectiveness of pavement texture recognition, ultimately contributing to safer and more sustainable road infrastructure.

## 2. Methodology

**Figure 1.**
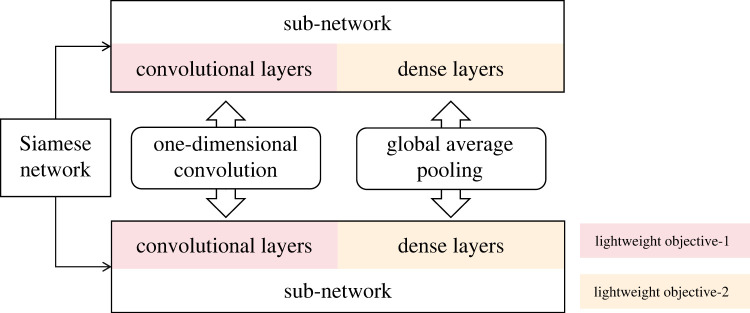
The flow chart of methodology.

This section presents the methodology employed in this paper for pavement texture recognition. First, the Siamese network is introduced, which serves as the few-shot learning network framework, highlighting the potential targets to lightweight in the Siamese network. Subsequently, alternative lightweight methods are discussed, including GAP, one-dimensional convolution and the integration of both. The figure 1 presents the basic structure and the lightweight routes of the Siamese network.

### (a) Siamese network architecture and lightweight objectives

In this study, the Siamese network was chosen for pavement texture classification, primarily due to its exceptional performance across various domains and its relatively simple architecture compared to other few-shot learning frameworks. The simplicity of the Siamese network makes it an appealing choice for addressing complex classification tasks while maintaining efficiency and ease of implementation. Furthermore, its demonstrated success in diverse applications [33–35] offers a strong foundation for adapting it to the specific requirements of pavement texture classification, ultimately contributing to the creation of more accurate and robust models. Although the Siamese network may entail higher computational demands when dealing with a larger number of categories, it remains applicable and well suited for the four-type pavement texture classification task addressed in this paper.

The Siamese network consists of two sub-networks with the same structure that share the same weights. These sub-networks generate embeddings for the input data, which are then fed to the energy function. The similarity of the input data is calculated based on the output of the energy function. For image classification, the Visual Geometry Group 16 (VGG16) was chosen as the sub-networks, which has been proven effective for extracting feature vectors in many studies based on Siamese networks [36–38].

**Figure 2.**
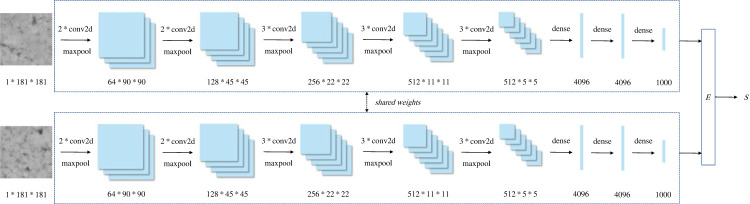
Original Siamese network.

Specifically, a pair of pavement texture images is input into the original Siamese network, with each image being processed by a separate sub-network. The sub-networks consist of a convolution layer, a maximum pooling layer and a dense connection layer, as shown in figure 2. The convolutional layers have a kernel size of 3 × 3 and channels of 64, 128, 256, 512 and 512, with the Relu activation function connecting each convolutional layer. The maximum pooling layers have kernels with a 2 × 2 size and strides with a 2 × 2 size. The first two dense connection layers have 4096 output nodes connected to the Relu activation function, while the last dense connection layer has 1000 output nodes connected to the Sigmoid activation function. The feature vectors output by the sub-networks are then used to calculate the energy function, which ultimately produces the similarity between the input images.

Regarding the lightweighting of the original Siamese network, the main targets are reducing storage volume and training time. The reduction of the number of parameters is the primary consideration for reducing the storage volume occupied by the classification model, which can also decrease the likelihood of overfitting. In terms of training time, the reduction of multiply-accumulate operations (MACC) in the network, a significant portion of computational overhead, is crucial. Table 1 shows the volume of parameters and MACC for the sub-networks in the original Siamese network. It is evident that the dense layers contain the majority of the parameters in the network, while the convolutional layers are the primary source of computational cost. Consequently, the lightweighting of the dense layers is crucial for reducing the storage volume of the network, and the lightweighting of the convolutional layers is essential for saving the training time.

**Table 1.** Parameters and MACC of the sub-networks of the original Siamese network.

no.	layer	output shape	parameters (×10^6^)	MACC (×10^9^)
1	conv2D	(64, 181, 181)	0.00064	0.019
2	conv2D	(64, 181, 181)	0.037	1.2
3	pooling	(64, 90, 90)	0	/
4	conv2D	(128, 90, 90)	0.074	0.60
5	conv2D	(128, 90, 90)	0.15	1.2
6	pooling	(128, 45, 45)	0	/
7	conv2D	(256, 45, 45)	0.30	0.60
8	conv2D	(256, 45, 45)	0.59	1.2
9	conv2D	(256, 45, 45)	0.59	1.2
10	pooling	(256, 22, 22)	0	/
11	conv2D	(512, 22, 22)	1.2	0.57
12	conv2D	(512, 22, 22)	2.4	1.1
13	conv2D	(512, 22, 22)	2.4	1.1
14	pooling	(512, 11, 11)	0	/
15	conv2D	(512, 11, 11)	2.4	0.29
16	conv2D	(512, 11, 11)	2.4	0.29
17	conv2D	(512, 11, 11)	2.4	0.29
18	pooling	(512, 5, 5)	0	/
19	flatten	(12 800)	0	/
20	dense	(4096)	52	0.052
21	dense	(4096)	17	0.017
22	dense	(1000)	4.1	0.00041
total parameters: 88 (×10^6^).				
total MACC: 9.8 (×10^9^)				

### (b) Lightweight method based on global average pooling

**Figure 3.**
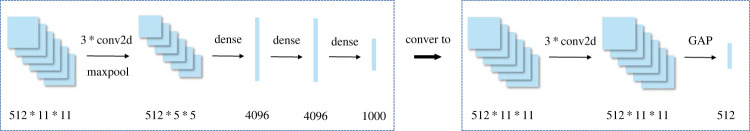
Lightweight for dense layers based on GAP.

GAP is a lightweight method used to reduce the number of parameters in dense layers [39]. It involves taking the average of all the pixels in the feature map of each channel, resulting in a single value per channel. The feature map is then transformed into a feature vector with a length equal to the number of channels. This method is useful in avoiding the high input dimensions that are usually required by dense layers. In this study, GAP is applied in place of dense layers, as illustrated in figure 3. This approach helps to reduce the number of parameters, storage volume and potential for overfitting.

### (c) Lightweight method based on one-dimensional convolution

**Figure 4.**
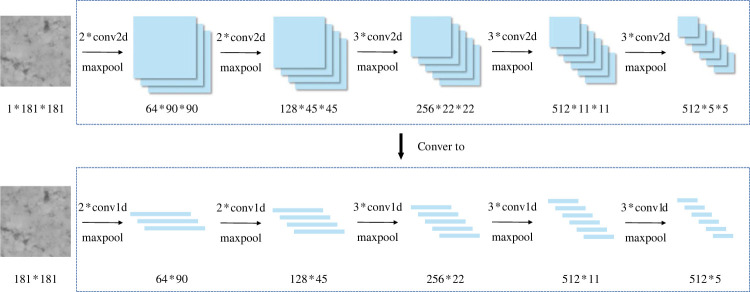
Lightweight for convolutional layers based on one-dimensional convolution.

One-dimensional CNNs are commonly used for processing text data [40]. However, given the simple characteristics of pavement texture, it is possible to perform pavement texture classification using one-dimensional convolution. Unlike two-dimensional convolution, one-dimensional convolution operates on feature vectors with only one dimension, and its convolution kernels also have only one dimension, significantly reducing the computational overhead and time cost. To accommodate one-dimensional convolution, the input of the sub-network is a 181-length vector with 181 channels, containing the same information as the original 181 × 181 single-channel image. Consequently, all two-dimensional convolution operations are replaced by one-dimensional convolution, and the pooling layers are updated to one-dimensional pooling, as illustrated in figure 4.

### (d) Lightweight method based on the integration of global average pooling and one-dimensional convolution

**Figure 5.**
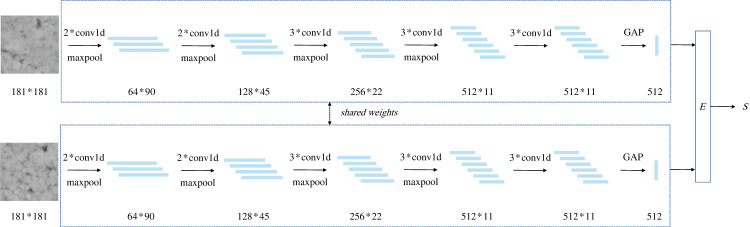
Lightweight Siamese network based on GAP and one-dimensional convolution.

The combination of GAP and one-dimensional convolution can yield an optimal lightweight effect. In this approach, the feature vector output of the last one-dimensional convolution layer is subjected to a GAP operation, where each dimension of the feature vector is averaged to obtain a scalar value. The resulting feature vector, which has a length equal to the number of channels of feature vectors generated in the last one-dimensional convolution layer, serves as the embedding that is fed into the energy function by the sub-network. The lightweight Siamese network structure that incorporates both GAP and one-dimensional convolution is illustrated in figure 5.

## 3. Experiment design

**Figure 6.**
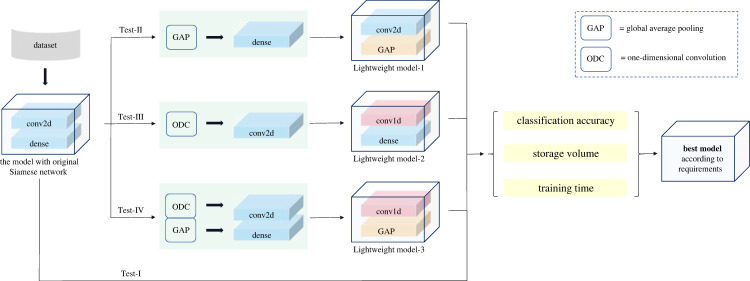
The flowchart of experiment design.

This section introduces the experimental design. First, it presents the pavement texture dataset used in this study. Subsequently, the design of the comparative experiments is discussed, including the specific network configurations and hyperparameters in the experiments designed to test the performance of the recognition models employing different lightweight methods. Finally, the experimental environment is described. The flowchart of the experiment is presented in figure 6.

### (a) Dataset of pavement texture

Three-dimensional pavement texture data were acquired using a laser scanner in Beijing, China, with a size of 90 × 90 mm and sampling intervals of 0.5 mm in two horizontal directions [41]. Each data was recorded as a 181 × 181 two-dimensional array with the corresponding height value. The height of the pavement texture was normalized prior to further processing, and the data were converted into grayscale images suitable for the application of image classification methods and visualization.

The classification task involves identifying four of the most common types of pavement texture: dense asphalt concrete (DAC), micro surface (MS), open-graded friction course (OGFC) and stone matrix asphalt (SMA). Typical images of each type of pavement texture refer to [11]. In this study, the training of the pavement texture recognition model was performed with a limited dataset of only five images per category. This constraint was motivated by the fact that collecting diverse and extensive samples of pavement texture images is a challenging and laborious process in road detection work. The main objective of this study was to propose a reliable model capable of achieving high accuracy in recognizing pavement textures despite the limited availability of training data, ultimately contributing to the overall efficiency and effectiveness of road surface analysis and maintenance. The number of images used for model training is presented in table 2. The proposed few-shot learning models were evaluated using a four-way five-shot classification task, which is typical in the field of few-shot learning [42–45].

**Table 2.** Sample composition of the dataset.

	DAC	MS	OGFC	SMA
sample size of training set	5	5	5	5
sample size of test set	45	4	14	45

### (b) Comparative experiments for evaluating the performances of the classification models

To reduce the storage volume and training time of the Siamese network and investigate how this affects the accuracy of the network in classifying pavement texture, a comparative experiment was designed. Various methods were applied to obtain lightweight models from the original Siamese network, which were then trained under the same conditions. The performance of the models with the original Siamese network and lightweight Siamese networks for pavement texture classification was compared comprehensively, considering their storage volume, training time and classification accuracy.

Specifically, four experiments were designed to evaluate the performance of the original Siamese network model and three lightweight models, as shown in table 3. The original Siamese network consisted of convolutional layers and dense layers, while the lightweight models were obtained by applying three different lightweight methods to the original Siamese network. Lightweight model-1 was obtained by replacing the dense layers of the original Siamese network with GAP. Lightweight model-2 used one-dimensional convolution for the convolutional layers in the original Siamese network. Lightweight model-3 applied GAP and one-dimensional convolution to the dense layers and the convolutional layers, respectively.

**Table 3.** Experiments using different lightweight methods of Siamese network.

		lightweight method	lightweight method for
Experiment	model	for dense layers	convolutional layers
Test-I	model with original Siamese network	/	/
Test-II	Lightweight model-1	GAP	/
Test-III	Lightweight model-2	/	one-dimensional convolution
Test-IV	Lightweight model-3	GAP	one-dimensional convolution

Prior to the training, sample pairs were constructed by pairing two pavement texture images and inputting them into the Siamese network. A sample pair consisting of two images belonging to the same category was defined as a positive sample pair, while a sample pair consisting of two images belonging to different categories was defined as a negative sample pair. All images in the training set were used to construct sample pairs. The number of positive and negative sample pairs was calculated using equations (1) and (2), respectively.

1 $$\begin{array}{rl} NUM_{p} & \;=N\cdot \frac{K!}{2(K-2)!}\\ NUM_{n} & \;=K^{2}\cdot \frac{N!}{2(N-2)!}, \end{array}$$

where *N* is the number of categories and *K* is the sample size of each category. In the pavement texture classification task of this study, the numbers of positive and negative sample pairs were 40 and 150, respectively. All the sample pairs were randomly divided into two equal parts for training and validation of the Siamese network. The statistics of sample pairs used for each experiment are presented in table 4.

**Table 4.** Sample pairs for each experiment.

		sample pairs		sample pairs partition	
		positive	negative	sample pairs	sample pairs
experiment	model	sample pairs	sample pairs	of training (%)	of validation (%)
Test-I	model with original Siamese network	40	150	50	50
Test-II	Lightweight model-1	40	150	50	50
Test-III	Lightweight model-2	40	150	50	50
Test-IV	Lightweight model-3	40	150	50	50

The Siamese networks used the Euclidean distance function as the energy function, which was defined as follows:

2 $$E=||x_{n}-z_{n}|{|}_{2},$$

where *x* and *z* represent the feature vectors output by the sub-networks and *n* is the dimension of the feature vector. The Energy function serves as a measure of the difference between samples. In particular, for a positive sample pair, the value of *E* will be very small, while for a negative sample pair, the value of *E* will be large.

The contrastive loss function was used to evaluate the match between E and the corresponding label, which can be defined as follows:

3 $$\text{Loss}-\frac{1}{2N_{s}}\sum_{i=1}^{N_{s}}yE^{2}+(1-y)\max{\left(m-E,0\right)}^{2},$$

where *y* represents the label of sample pairs, *1* for positive sample pairs and *0* for negative sample pairs, *N_s_* is the number of samples, and *m* is a threshold set to 1 in this paper. If the Siamese network is effective, the value of *E* for positive sample pairs tends to approach *0*, while the value of *E* for negative sample pairs is significantly larger, resulting in a low value of *Loss*. Conversely, if the value of *E* for positive sample pairs is large and that for negative sample pairs is close to *0*, the *Loss* value will be high.

The RMSprop optimizer was used with a learning rate of 10^−5^, and the training epochs were set to 20 with a batch size of 16. The other parameters were set to their default values; each experiment was repeated five times under the same conditions.

### (c) Experimental environment and dependent equipment

The experiments were performed on a laptop with an Intel Core i9-9880H CPU@2.30GHz, 16 cores and 32GB RAM. All models were implemented in Python using the TensorFlow deep learning library.

## 4. Result and discussion

### (a) Comparison of pavement texture classification accuracy amongthe lightweight models

**Figure 7.**
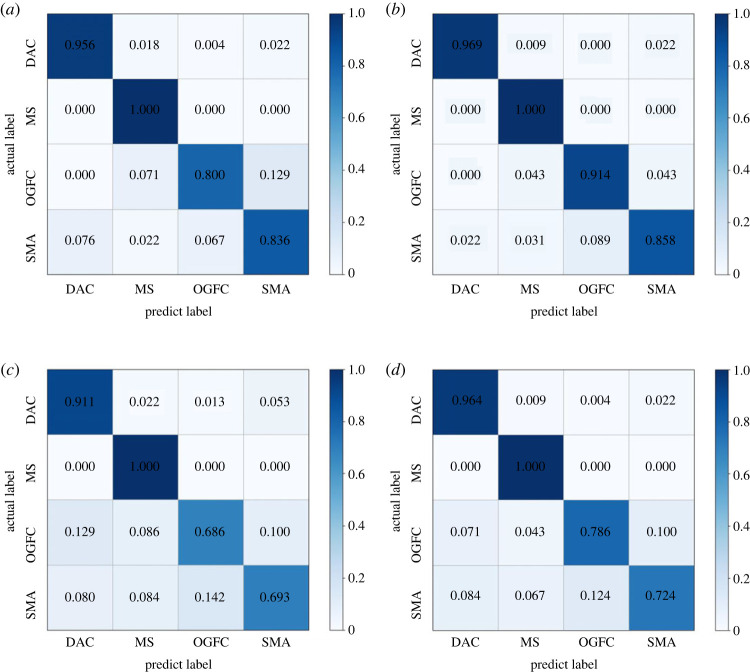
Classification confusion matrix of each experiment. (*a*) Test-I, (*b*) Test-II, (*c*) Test-III and (*d*) Test-IV.

The Siamese network can only infer similarity between two input images and cannot directly classify them into specific categories. Therefore, the comparison verification method was used to classify pavement texture images in the test set. Specifically, for each image in the test set, sample pairs were constructed using five images of each category in the training set and input into the trained Siamese networks. The energy function values of the test image and each category of images in the training set were averaged to obtain the differences between the test image and various pavement textures. The category with the least difference from the test image was considered as the classification result. The classification confusion matrix of each experiment is presented in figure 7. The high classification accuracy for each category of pavement texture in each experiment indicates that the original Siamese network and each lightweight Siamese network were effective.

The comparison of classification accuracies is presented in table 5, where the average accuracy was defined as the arithmetic average of accuracy in each category. It was observed that Lightweight model-1 achieved the highest average accuracy, which could be attributed to the GAP method that helped alleviate the overfitting problem during the training process. On the other hand, the average accuracies of Lightweight model-2 and Lightweight model-3 were lower than that of the Original Siamese network, indicating that one-dimensional convolution was less effective for classification than two-dimensional convolution. Nevertheless, all models proposed in this study achieved an average accuracy higher than 80%, thus indicating their effectiveness for pavement texture classification. Notably, the MS classification accuracy was 100% due to its significant differences from other categories, while the OGFC and SMA classification accuracies were low as they were similar to each other.

**Table 5.** Comparison of classification accuracies.

		classification accuracy on test set (%)				
experiment	model	DAC	MS	OGFC	SMA	average
Test-I	model with original Siamese network	95.6	100	80.0	83.6	89.8
Test-II	Lightweight model-1	96.9	100	91.4	85.8	93.5
Test-III	Lightweight model-2	91.1	100	68.6	69.3	82.3
Test-IV	Lightweight model-3	96.4	100	78.6	72.4	86.9

### (b) Comparison of lightweight degree among the lightweight models

The degree of lightweight of a network is a critical factor in practical engineering. As the major indicator of network lightness, storage volume was compressed by reducing parameters and training time was saved by reducing MACC in this study. The degree of lightness of the proposed models is summarized in table 6. Compared to the original Siamese network-based model, the storage volume of Lightweight model-1 and Lightweight model-3 was substantially reduced due to GAP, which decreased dense layer parameters. Lightweight model-2 and Lightweight model-3 were superior in terms of training time, as one-dimensional convolution actively reduced the MACC of the convolutional layer. Overall, Lightweight model-3 achieved the most desirable lightweight effect, with the lowest storage volume and shortest training time.

**Figure 8.**
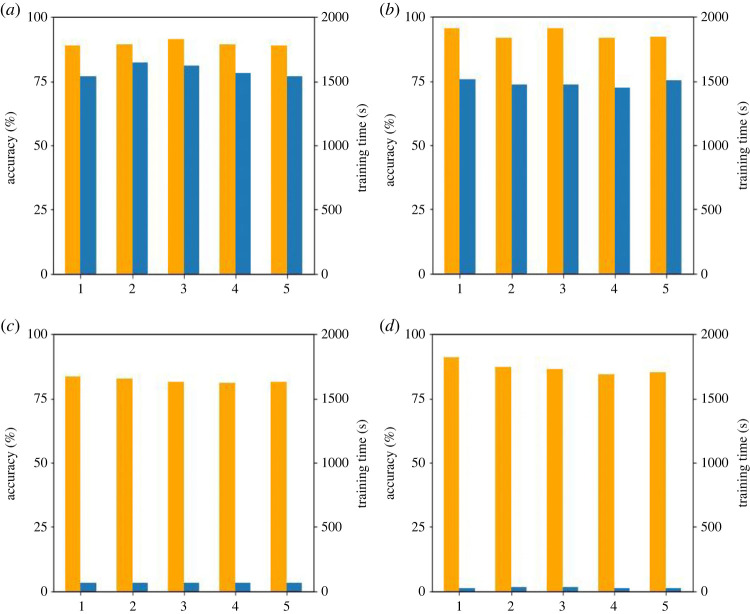
Accuracy and training time of each experiment repeated five times. (*a*) Test-I, (*b*) Test-II, (*c*) Test-III and (*d*) Test-IV.

**Table 6.** Indicators of network lightness of the proposed models.

		parameter quantity	storage volume	MACC	training
experiment	model	(×10^6^)	(MB)	(×10^9^)	time (s)
Test-I	model with original Siamese network	88	336	9.8	1583
Test-II	Lightweight model-1	15	56	9.7	1484
Test-III	Lightweight model-2	36	139	0.18	65
Test-IV	Lightweight model-3	4.9	19	0.11	27

Before proceeding further, it is important to note that the aforementioned data is reliable. The parameters quantity and MACC are considered to be theoretical values, whereas storage volume, training time and accuracy are experimental values that require rational analysis. Based on the results obtained after each experiment was repeated five times, it was observed that the storage volume remained constant, and the accuracy and training time remained mostly stable, as depicted in figure 8. Furthermore, calculations revealed that the accuracy and training time for each experiment fell within twice the s.d. range with no outliers detected.

**Table 7.** Comprehensive comparison of Siamese networks.

	parameter quantity		storage volume		MACC		training time		test accuracy	
model	(×10^6^)	reduction (%)	(MB)	reduction (%)	(×10^9^)	reduction (%)	(s)	reduction (%)	(%)	increase (%)
model with original Siamese network	88	/	336	/	9.8	/	1583	/	89.8	/
Lightweight model-1	15	83	56	83	9.7	1	1484	6	93.5	3.7
Lightweight model-2	36	59	139	59	0.18	98	65	96	82.3	−7.5
Lightweight model-3	4.9	94	19	94	0.11	99	27	98	86.9	−2.9

### (c) Comprehensive analysis of classification accuracy and lightweight degree

A comprehensive comparison of model performances is presented in table 7. The experimental results of the Original Siamese network were used as the benchmark for comparison. Lightweight model-1 achieved the highest classification accuracy of 93.5%, which was 3.7% higher than the Original Siamese network. Importantly, the accuracy increase referred to the absolute increase in this paper. In addition, Lightweight model-1 significantly compressed the storage volume by 83% and saved 6% training time. For the lightweight effect, Lightweight model-3 performed the best, compressing 94% of the storage volume compared to the model with the original Siamese network and taking only 27 s to complete the training. However, the classification accuracy of pavement texture based on Lightweight model-3 was reduced by 2.9%.

In essence, GAP significantly reduced the storage volume by replacing dense layers, while one-dimensional convolution also achieved some effect by reducing the number of input nodes in dense layers. Moreover, one-dimensional convolution drastically cut the training time of the Siamese network by reducing the MACC of the convolutional layers. However, GAP had little effect since the dense layers only account for a small fraction of the MACC of the network. It should be noted that the reduction rate of storage volume and parameter quantity was the same since the network saved only parameters when storing. However, the reduction rate of training time was different from the reduction rate of MACC due to reasons such as the amount of computation of the activation function not being counted. Additionally, GAP improved accuracy slightly, while one-dimensional convolution had a slight effect on reducing accuracy.

## 5. Conclusion

This study proposes a few-shot learning approach based on a Siamese network for classifying pavement texture with a limited dataset. To enhance the practical application of the models, lightweight methods are incorporated into the Siamese network. Comparative experiments are conducted to evaluate the performance of the original Siamese network and the lightweighted Siamese networks in a four-way five-shot pavement texture classification task. The findings indicate that (i) all models achieve over 80% accuracy, confirming the feasibility and effectiveness of few-shot learning for pavement texture classification. Moreover, implementing lightweight methods reduces the storage volume and training time of the models without compromising accuracy. (ii) The model that applies GAP on the dense layers of the Siamese network attains a classification accuracy of 93.5% while reducing storage volume by 83% and saving 6% of training time, thus serving as a suitable choice for high-accuracy classification tasks. (iii) The lightweight model that uses one-dimensional convolution on convolutional layers and GAP on dense layers may be more appropriate for tasks that prioritize classification speed and storage volume, with only 19 MB of storage requirement and less training time.

As an initial exploration of pavement texture classification using few-shot learning, this study has several limitations that warrant acknowledgement. First, the study only investigated the use of Siamese networks for constructing few-shot learning models. Other network architectures that implement few-shot learning may offer advantages over Siamese networks and should be considered for further investigation. Second, the dataset used in this study is limited due to the difficulty of obtaining pavement texture images, which constrained the types of pavement textures considered in the recognition model. Considering the diverse range of pavement textures and the potential variability within the same type of pavement surface, future research should focus on expanding the applicable types of textures and improving recognition accuracy with larger datasets. Finally, there may be additional approaches for creating lightweight pavement texture classification models beyond the two methods employed in this study. Future research should investigate the development of lightweight models that both improve accuracy and significantly reduce storage volume and training time.

## Data Availability

The test/train dataset used in this study is available as supplementary material [46].
